# Using Information Videos to Improve Patient Satisfaction in Endoscopy: A Prospective Service Improvement Project

**DOI:** 10.7759/cureus.24108

**Published:** 2022-04-13

**Authors:** Ephraim Broder, Amelia Davies, Laith Alrubaiy

**Affiliations:** 1 Gastroenterology, Imperial College London University, London, GBR; 2 Gastroenterology, London North West University Healthcare NHS Trust, London, GBR

**Keywords:** video feedback, endoscopy intervention, patient consent, patient counseling, gastroenterology and endoscopy

## Abstract

Background: Endoscopy is a rapidly developing discipline with new techniques and procedures being introduced each year. The consenting process is central to patient perception; using information videos as additional tools to aid consent and improve the quality of care in endoscopy is not well established. Our aim was to develop, implement and validate the use of patient educational videos to improve patients’ satisfaction and experience in endoscopy.

Methods and analysis: This was a prospective service quality improvement study. Eligible patients were invited to watch the educational videos in addition to standard practice. We compared this group with a matched cohort of patients who were receiving standard care of postal information leaflets. Patient satisfaction was measured through the validated Gastrointestinal Endoscopy Satisfaction Questionnaire (GESQ).

Results: Patients in the intervention group scored four questions relating to pre-procedural information significantly higher than the control (p=0.038). The total mean GESQ score was also higher in the intervention group compared to the control, however this was not statistically significant (p=0.397). The intervention group had significantly lower cancellation rate (4%) compared to the control group (20%), p=0.023.

Conclusions: Patients who watched educational videos were more satisfied with pre-procedural information in the consenting period than those who did not. Further research is still needed to determine if they reduce patient anxiety. Meanwhile, it would be appropriate to implement these videos into routine practice as a cost-effective method of improving patient satisfaction.

## Introduction

Oesophago-gastro-duodenoscopy (OGD) and colonoscopy have been shown to be associated with high levels of pre-procedure anxiety [1]. The predominant causes of anxiety focus on fears about bowel preparation, pain during the procedure, the effects of sedation and worries about potential complications [2,3]. Although some of these fears are difficult to mitigate, some are due to lack of information. Therefore, it is important to provide clear and correct pre-procedural information to improve patient satisfaction.

Visual aids are currently under-utilised to convey information to patients and consolidate the consenting process [4]. Visual aids and high-quality infographics have an advantage over script in mitigating language barriers and comprehension issues.

Using videos directed at patients to improve their understanding of endoscopy procedures is not well established. Although visual resources are available on the internet, they are not validated. Luck et al. (1999) assessed how anxiety is affected by pre-procedural informational videos [5] but with the advancements in technology and growing proportion of tech-savvy patients, there has been little further assessment of educational videos in endoscopy care.

The aim of the study was to develop, implement and validate short patient educational videos about endoscopy procedures and assess the effect on patient satisfaction compared with the current standard of care.

## Materials and methods

Study design

This was a prospective service quality improvement study conducted at the Wolfson Unit for Endoscopy at St Mark’s Hospital, North West London. The study looked at a group of patients who, in addition to receiving the standard information leaflets, were also invited to watch endoscopy educational videos prior to their procedure. They were compared to a control group who received standard care.

Patient selection

A convenience sample was taken from five endoscopy lists. All patients who were scheduled for an OGD or colonoscopy between March - April 2021 were reviewed for inclusion in the study. Patients were then contacted via telephone for further assessment and consenting. Those that met the inclusion criteria underwent stratified randomisation into the Intervention group and Control group. Inclusion and Exclusion Criteria can be see in Table 1. There was no blinding of investigators.

**Table 1:** Inclusion and Exclusion Criteria

Inclusion Criteria	Exclusion Criteria
Patients who are going to have a diagnostic OGD or colonoscopy procedure	Patients who are unable to comprehend the study
Aged 18 to 90	Patients unable to access the videos due to cultural or technical barriers
Native or fluent English speaker	Patients who have previously undergone endoscopy
	Vulnerable group of patients
	Visual impairment
	Patients who are having emergency or lifesaving endoscopy procedures
	Patients unable to consent
	Severe co-morbidities and poor performance status

Baseline data for all patients was collected upon recruitment

Baseline data for all patients was collected. This included socio-demographic data (age, sex and ethnicity), administrative data (type of procedure) and clinic data on the patients co-morbidities and indication for endoscopy. 

Intervention

A focus group of endoscopy consultants, nurses and patient representatives met to identify the key material that needed to be covered within the educational videos. Procedure-specific short video clips were developed in liaison with an on-site video producer. The videos included a clear explanation of the preparation before the procedure, the procedure itself, what to expect on the day, the follow-up arrangements and contact information. Videos were hosted on YouTube [6-12]. 

Due to COVID-19 infection control measures, videos and surveys were emailed to participants. The intervention group were sent links to the YouTube videos via email along with a short explanation of the purpose of the videos and a request for feedback. 

The emails were sent at least one week prior to the procedure. Those who declined using email were given the option to receive the YouTube links via text message instead. 

This project used a validated and newly developed patient stratification questionnaire, Gastrointestinal Endoscopy Satisfaction Questionnaire (GESQ) [13]. Participants were emailed the GESQ two days after their procedure. The responses were collected using Google Forms survey and unique identification numbers to ensure patient confidentiality.

Outcome measures

Outcome measures were: 1. Patient-specific: Patient satisfaction was measured using GESQ; 2. Procedure-specific: Number of last-minute cancellations; 3. Potential problems that may be encountered in using videos to supplement the consenting process; and 4. Transferable new knowledge about the challenges of integrating this service model in health care organisations.

Statistical analysis

Data was collated in Microsoft Excel (Microsoft, Redmond, WA, USA) and survey responses were coded. Responses “very easy”, “easy”, “fair”, “difficult” and “very difficult” were coded as 4, 3, 2, 1 and 0 respectively. Questions that were negatively worded were reversely coded. The scores for each participant were added up to give a total score. A higher score corresponded to a greater overall satisfaction. 

The mean scores of the intervention group and the control group were then compared using the Mann-Whitney U Test. Sub-group analysis was also carried out on individual questions specifically relating to the usefulness and clarity of the information given prior to the endoscopy. Fisher exact test was used to compare cancellation data due to small numbers. All statistical analysis was performed using SPSS v 27.0 (IBM Corp., Armonk, NY, USA).

Ethics and dissemination 

The study was a service quality improvement project. The videos were presented to patients and expert focus groups to ensure they were acceptable to patients. Verbal consent was gained from all study participants. Since no patient identifiable information was shared, and the management of individual patients was not affected, the study did not require ethics approval by the NHS Health Research Authority.

## Results

**Figure 1:**
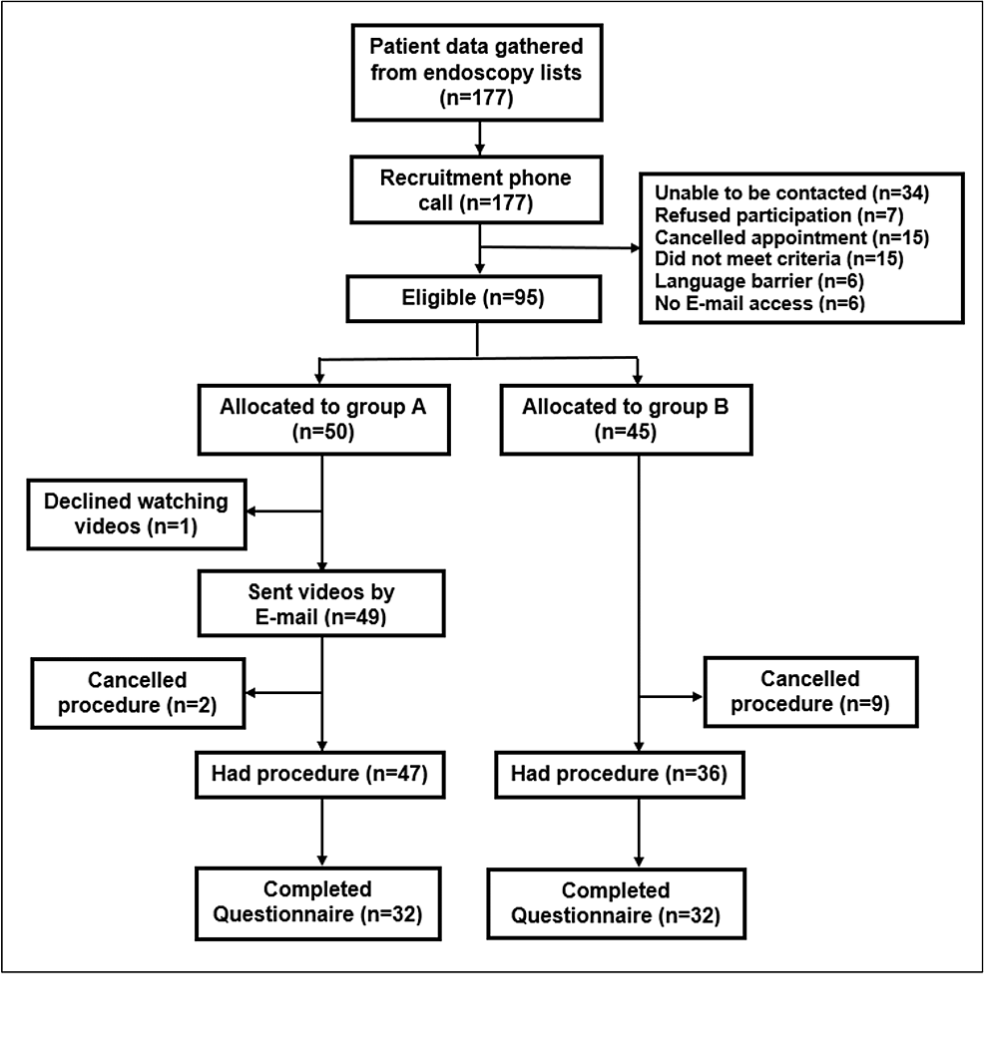
Flowchart showing the recruitment process 177 patients were initially contacted. Out of the 95 eligible patients, 50 were allocated to Group A (intervention group shown the videos) and 45 to Group B (control group). After having the procedure 32 patients from each group completed the online survey. Patients were lost at the initial recruitment (n=82), refusing to watch videos (n=1) and cancelled procedures (n=11).

One hundred seventy-seven patients who were due to have an OGD or colonoscopy during the recruitment period were contacted. Of those who answered the phone, 95 met the criteria and consented. A flowchart showing the recruitment process is shown in Figure 1. 

The patients who completed the questionnaire in the intervention group and control group were stratified for age (54.5 years [intervention group], 55.1 years [control group]), age range (18 - 84 years [intervention group], 19 - 83 years [control group]) and sex (male 31.3% [intervention group], 21.9% [control group]). The two groups also had a similar proportion of patients undergoing OGDs and colonoscopies. Two patients in the intervention group and one in the control group had combined procedures. A summary of the patient characteristics can be seen in Table 2. 

**Table 2:** Patient characteristics of the intervention group (n=32) and control group (n=32) OGD; Oesophago-Gastro-Duodenoscopy

	Intervention Group (n=32)	Control Group (n=32)
Age		
Mean ±SD	54.5 ± 18.0	55.1 ± 17.4
Sex, n (%)		
Male	10 (31.3)	7 (21.9)
Female	22 (68.7)	25 (78.1)
Procedure, n (%)		
OGD	20 (62.5)	22 (68.8)
Colonoscopy	10 (31.3)	8 (25)
OGD + Colonoscopy	2 (6.2)	1 (3.1)

The primary outcome of the study was patient satisfaction, which was quantitively measured using the GESQ. The intervention group was found to have a higher mean score, 59.6 compared to 57.3, although this was not found to be statistically significant (p=0.397). 

Questions 1, 2, 4 & 5 from the GESQ focused on patients’ satisfaction with pre-procedural information. Patients in the intervention group had a higher mean score in these four questions. The total sub-score of these questions was significantly higher in the intervention group, with a mean of 13.8 compared to 12.3 (p=0.038).

**Figure 2:**
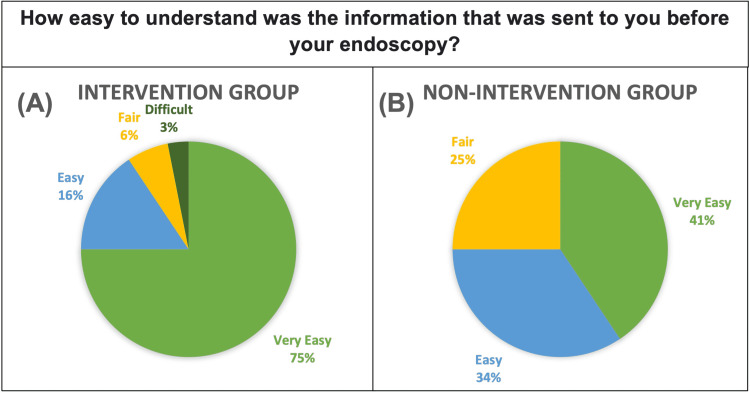
Pie Chart showing responses of Intervention group. (B) Pie Chart showing responses of Non-Intervention group. Response choices were “Very Easy”, “Easy”, “Fair”, “Difficult” and “Very Difficult”.

When asked about the ease of understanding the information sent prior to the endoscopy (either information leaflets or information leaflets + videos), 75% of the intervention group selected “Very Easy” compared to 41% of the control group. This can be seen graphically in Figure 2. The mean score for this question was significantly higher for the intervention group (p=0.019).

**Figure 3:**
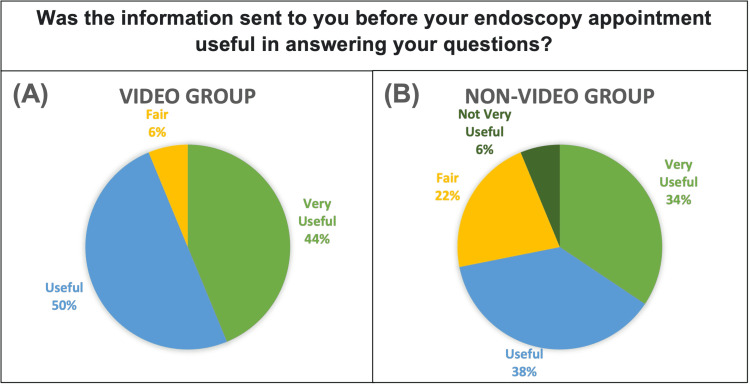
Pie Chart showing responses of Video Group. (B) Pie Chart showing responses of Non-Video Group. Response choices were “Very Useful”, “Useful”, “Fair”, “Not Very Useful” and “Not at all Useful”.

When asked about the usefulness of the information they received pre-procedure, 94% of the intervention group answered “Useful” (50%) or “Very Useful” (44%). Whereas the control group showed great range with “Not Very Useful” (6%), “Fair” (22%), “Useful” (38%) and “Very Useful” (34%). This can be seen in Figure 3. On average, the intervention group found the pre-procedure information significantly more useful (p=0.049).

**Figure 4:**
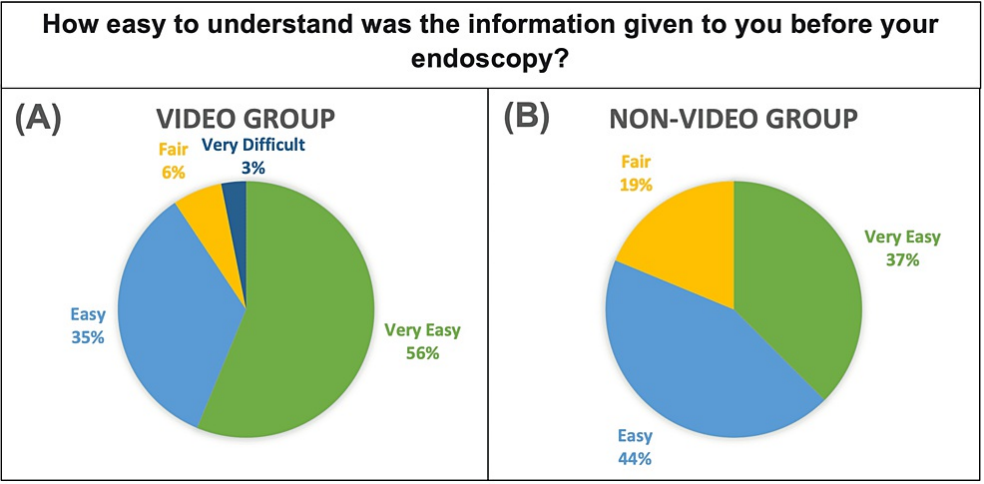
Pie Chart showing responses of Video Group. (B) Pie Chart showing responses of Non-Video Group. Response choices were “Very Easy”, “Easy”, “Fair”, “Difficult” and “Very Difficult”.

**Figure 5:**
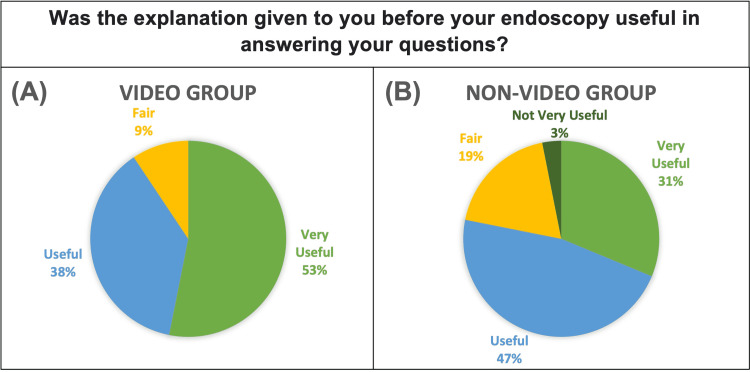
Pie Chart showing responses of Video Group. (B) Pie Chart showing responses of Non-Video Group. Response choices were “Very Useful”, “Useful”, “Fair”, “Not Very Useful” and “Not at all Useful”.

Questions 4 and 5 asked about the usefulness and ease of understanding of the explanation given prior to the procedure. As shown in Figure 4 and Figure 5 respectively, the intervention group scored both questions higher than the control group. This difference was significant for usefulness (p=0.044), but not for ease of understanding (p=0.284).

In the intervention group there were only two cancellations or non-attendees out of 49 patients compared to nine out of 45 patients in the control (p=0.023).

As well as the quantitative data gathered from the responses to the GESQ, individual qualitative feedback was welcomed. Three patients sent feedback via email. All feedback on the videos was positive with some suggestions for improvements. The videos were described as “clear and concise”, “easy to understand” and “had really good titbits of key information”. One patient wrote her questions concerning sedation had been satisfactorily answered after watching the videos. Recommendations from the patients included adding non-English subtitles, adding more information on the foods able to be eaten prior to endoscopy and having more patients from the black, Asian, and minority ethnic (BAME) community represented in the videos. 

## Discussion

The results of the study showed the pre-endoscopy videos to be effective at improving patient experience and satisfaction. The total mean GESQ score was higher in the intervention group but was not statistically significant (p=0.397). Whereas the sub-score for questions relating to pre-procedural information was significantly higher in the intervention group compared to controls (p=0.038). The four questions contributing to this sub-score were the most relevant to determining the effectiveness of the videos. The successful use of the videos was further reinforced by the positive feedback volunteered by participants who watched them. These findings were in line with previous studies [14-16].

The complete scope of the GESQ was broad, with many of the questions about external factors such as the comfort of the waiting room and the reputation of the hospital. This could account for the discrepancy between the significance of the total GESQ score and the sub-score as watching videos beforehand was not predicted to increase satisfaction in these areas. Furthermore, the sample size was relatively small (n=64), reducing the power of this study. 

Overall, the responses were consistently positive, with <5% negative responses to any of the questions across both groups. This indicates a high baseline level of patient satisfaction with the endoscopy procedures at St. Mark’s Hospital. 

We found a lower cancellation/did not attend (DNA) rate for the intervention group (4% compared to 20%), suggesting the videos answered questions and allayed patient fears. Studies have found endoscopy non-attendance rates to be between 14.7% to 29% [17-19]. Lowering these rates is important for reducing harm to patients and cutting costs for the NHS [20]. We were unable to record the reasons for cancellation and so factors such as bowel preparation issues and COVID-19 infection control may have played a role [2,21].

Strengths and limitations

Due to new infection control measures, videos and feedback were provided to patients via email. Although electronic surveys often have a lower response rate compared to postal surveys [22], the method employed in this study of individual phone calls and personalised email effectively demonstrated a high response rate. 

Despite the effective model used, the study had several limitations. The study was exploratory, so no pre-calculated sample size nor randomisation was used. The final groups were well matched in age, sex and type of procedure. The study excluded non-English speakers and those technically unable to watch the videos. These patients may also struggle with the current service of written information sent to them and so further study is needed to address the barriers for these vulnerable patients. Furthermore, as with any study conducted using questionnaires, there was a potential for response bias, however this was controlled as best as possible by having equal number of responses for both control and intervention groups. 

One patient in the intervention group refused to watch the videos out of fear of further increasing their anxiety. Implementation of video information would not disadvantage patients as they can simply refrain from watching the videos. However, we were unable to address whether there may be another subset of patients who believe they would benefit from the videos, only to become more nervous as a result of watching them. The feedback on the videos needs to be carefully monitored to be able to optimise the level of information delivered. 

This study has proved the efficacy of these specific videos prior to OGD and colonoscopy. The results cannot necessarily be extrapolated to using other videos or replicating them for other procedures. However, the elements included in the videos produced for this study (the preparation before the procedure, the procedure itself, what to expect on the day, the follow-up arrangements and contact information) can set a precedent for those trying to produce similar videos for other procedures. Endoscopy is also a particularly frightening and unknown procedure for patients compared to other procedures [1,23]. Therefore, each procedure needs to be considered on a case-by-case basis whether it is worth creating and implementing informational videos.

## Conclusions

This study adds further support to the current literature on providing visual information to patients prior to endoscopy. This study indicates that it would be beneficial to provide these videos as part of the routine information received prior to an endoscopy procedure in order significantly improve patient satisfaction. Producing the videos would be a cost-effective method of disseminating information to patients, which can be easily updated to sustain its relevancy.

While this study does show that educational videos improve patient satisfaction with pre-procedural information as well as reduce cancellations, it remains unclear whether they are effective at reducing anxiety. Further work is needed to assess whether videos can lower levels of anxiety.

Overall, endoscopy information videos were well-received and patients found them useful and easy to understand. It would be appropriate to implement these videos into routine practice while constantly monitoring patient feedback and updating them as necessary.
